# Microscopic Analysis of Steel Corrosion Products in Seawater and Sea-Sand Concrete

**DOI:** 10.3390/ma12203330

**Published:** 2019-10-12

**Authors:** Gang Wang, Qing Wu, Xue-Zhong Li, Jun Xu, Yao Xu, Wen-Hao Shi, Shi-Lin Wang

**Affiliations:** 1 College of Civil Engineering and Architecture, Jiangsu University of Science and Technology, Zhenjiang 212000, China; wgstarsky@163.com (G.W.); wuqing@just.edu.cn (Q.W.); fxxuyao@163.com (Y.X.); 15751774639@163.com (W.-H.S.); Shilin_Wang0602@126.com (S.-L.W.); 2 Graduate School of Sciences and Technology for Innovation, Yamaguchi University, Ube, Yamaguchi 755-8611, Japan; 18751800893@163.com; 3 School of Materials Science and Engineering, Southeast University, Nanjing 211189, China

**Keywords:** seawater and sea-sand concrete, steel corrosion products, XRD, SEM

## Abstract

In this paper, the types, formation time, structural morphology, and influence of steel corrosion products in seawater and sea-sand concrete were studied, and the intermediate and final products of steel corrosion under different conditions were determined. The corrosion products of steel in these concrete specimens under two curing methods were studied separately by X-ray powder diffraction (XRD) and scanning electron microscopy (SEM). Due to the presence of a large amount of chloride ions in the concrete, the rust layer on the surface of a steel bar contained many intermediates, such as lepidocrocite (γ–FeOOH) and aka-ganeite (β–FeOOH). Under wet/dry cycles, with the addition and loss of moisture in concrete, various corrosion products were also dynamically converted into each other. In the specimens immersed in seawater for a long time, the intermediates of corrosion were lepidocrocite (γ–FeOOH) and aka-ganeite (β–FeOOH), which were substituted for oxygen as the new depolarizers of cathode reduction reaction, and consumed themselves to ensure smooth corrosion.

## 1. Introduction

With the continuous development of marine resources in China, the construction of islands is an unavoidable topic. Considering the cost of transportation and time, it is of great practical significance to use sea stones and sea-sand on reefs as concrete aggregates for materials in island engineering projects. Due to the large amount of chloride adhering to the aggregate surface of seawater and sea-sand concrete, the steel reinforcement in concrete is the first to be affected by corrosion [1,2,3,4,5,6].

For a long time, researchers have extensively studied the corrosion of steel bars in concrete caused by chloride ions, but most of the studies are focused on the corrosion rate of steel bars and the degradation of the structural properties of reinforced concrete [7,8,9,10,11,12]. The corrosion composition of low carbon steel and weathering steel in atmospheric environment was also analyzed [13,14,15,16]. Researchers have also investigated the formation mechanism of various corrosion components of climate steel bars in atmospheric environment [17,18,19,20,21].

Refait et al. [22] found that in the presence of chloride ions, ferrous hydroxide reacts with chloride first to produce a very unstable green rust hydrochloride ${\mathrm{GRCl}}^{-}({\left[{\mathrm{Fe}}_{3}^{2+}{\mathrm{Fe}}^{3+}{\left({\mathrm{OH}}^{-}\right)}_{8}\right]}^{+}\cdot {\left[{\mathrm{Cl}}^{-}\cdot {\mathrm{nH}}_{2}O\right]}^{-}$ containing ferrous and ferric iron, and then it continues to oxidize into (β–FeOOH) or (γ–FeOOH) as an intermediate transition product. Legrand et al. [23] established that in the presence of ${\mathrm{SO}}_{4}^{2-}$, ferrous hydroxide reacts with sulfate to form green rust sulfate ${\mathrm{GRSO}}_{4}^{2-}({\left[{\mathrm{Fe}}_{4}^{2+}{\mathrm{Fe}}_{2}^{3+}{\left({\mathrm{OH}}^{-}\right)}_{12}\right]}^{2+}\cdot {\left[{\mathrm{SO}}_{4}^{2-}\cdot 2H_{2}O\right]}^{2-}$) first, and then continues to oxidize to (γ–FeOOH) as an intermediate transition product. The ferric hydroxide produced by the corrosion of carbon steel can be divided into different crystal types. At the initial stage of corrosion, the products are mainly (γ–FeOOH).

Although there are many studies on the effect of chloride ion on steel bars at home and abroad, in order to shorten the test period, most of them are simulated experiments of concrete simulated pore solution, accelerated corrosion of steel under current and artificial cracking. The study designs are single, and most of them investigated macro-mechanical properties, without the use of XRD, SEM, and other means from the micro perspective. However, the studies on phase composition and microstructure of rust layer in concrete are still lacking in systematic theory, which cannot accurately describe the mechanism of steel corrosion in seawater sand concrete. Therefore, the research on this aspect needs to be improved.

In view of the main problems in the durability study of seawater and sea-sand concrete at present, several seawater and sea-sand concrete specimens and ordinary concrete specimens were prepared in this study, to simulate the corrosion of steel bar specimens under the conditions of continuous immersion in marine environment and dry-wet cycling, respectively, in order to study the corrosion of steel bar in the specimens. Scanning electron microscopy (SEM) was used to photograph and analyze the surface of steel bar rust layer, and the types, generation time and effect on steel bar matrix were judged according to the morphology of rust products. X-ray diffraction was used to obtain the diffraction patterns of corrosion products of different rust layers of steel bar, and the composition and internal structure of the products were obtained. According to the environment of the specimens, the main intermediate products of steel corrosion and the final oxidation products under different conditions were deduced, and the complex transformation relationship between various corrosion products was determined.

## 2. Experimental Program

### 2.1. Preparation of Specimens

Cylindrical specimens with dimensions of Φ × L = 52 mm × 320 mm were adopted to ensure that the corrosion of the embedded steel bar in all directions was identical. The cement used was P.O32.5. In the seawater and sea-sand concrete, sea stones, and sea-sand were used for aggregates, but ordinary gravel and natural medium sand were used in ordinary concrete. The fine modulus $M_{x}$ of natural medium sand and sea-sand are 2.8 and 2.65, respectively, and the chloride ion content of sea-sand is 0.24%. The mixing proportion of the specimen is m(cement):m(water):m(sand):m(stone)= 1:0.41:1.05:2.58.

**Figure 1:**
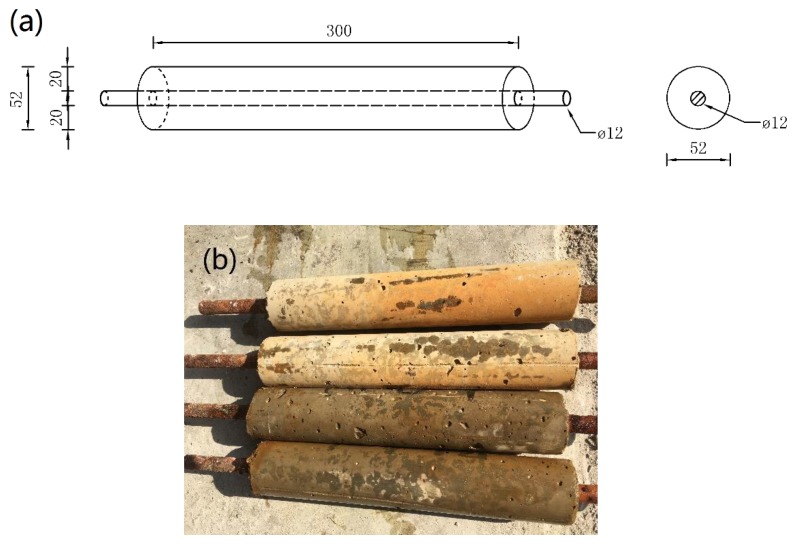
Details of test specimen; (**a**) Schematic diagram, (**b**) Photograph.

The steel bars used in the specimens were all made of the same batch of HPB235 ordinary low carbon steel with a diameter of 12 mm. The composition of the steel bars is shown in Table 1. Each steel bar was 320 (±1) mm in length, and there were no rust pits and other major defects on the surface. The steel bar was immersed in 12% hydrochloric acid solution, rinsed with clean water, neutralized with lime water, rinsed with clean water, and placed in the dryer for 4 h. The bare part of the steel bar was coated with anti-rust paint to ensure its non-corrosion. The schematic and physical drawings of the specimens are shown in Figure 1.

### 2.2. Specimen Maintenance Method

**Figure 2:**
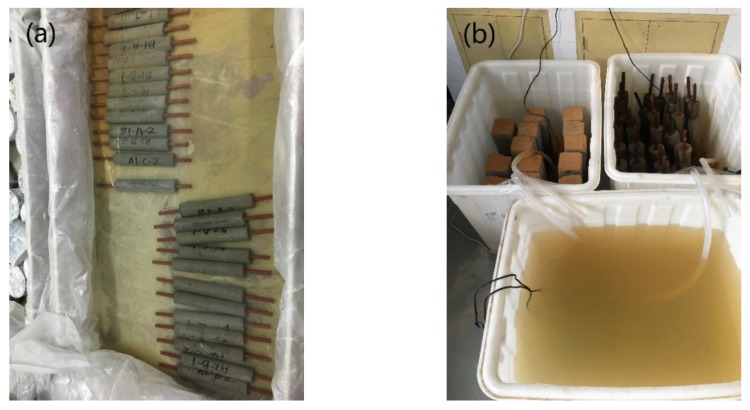
Images of curing setup for test specimens; (**a**) Setup of the immersion process, (**b**) Setup of the wet/dry cycles.

After the specimens were made, they were placed in the curing room in different groups. One group was immersed continuously in artificial seawater to simulate the underwater environment of the ocean. The other group used a wet/dry cycles device to simulate the tidal environment of the ocean. The specimens were immersed for two days and dried for two days. The water temperature was 20 ± 5 °C (Figure 2). The grouping of specimens is shown in Table 2.

### 2.3. Preparation and Observation of Samples

#### 2.3.1. XRD Analysis

**Figure 3:**
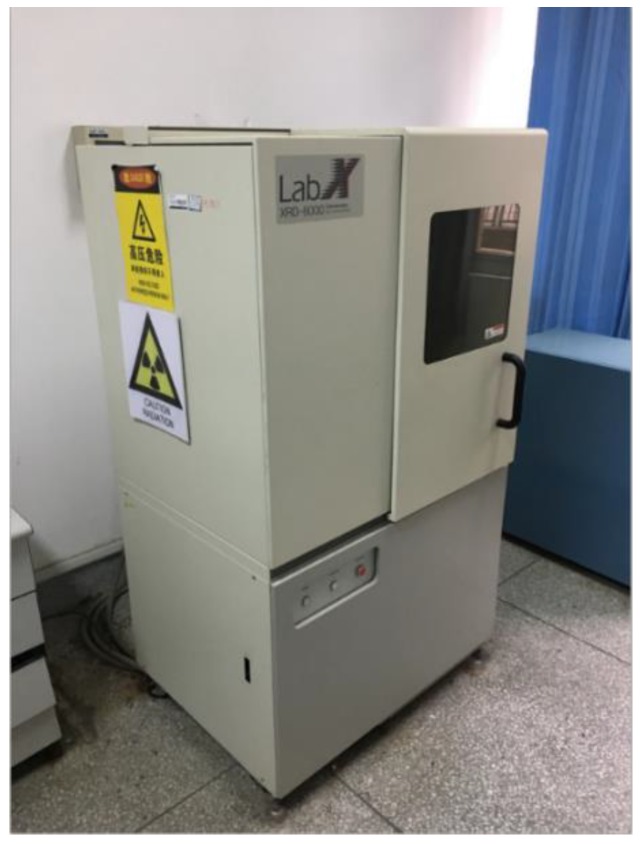
XRD-6000 model X-ray powder diffractometer.

First, the corroded steel bars in concrete were taken out. The corrosion layers can be divided into internal rust layer and external rust layer according to the structure. The external rust layer was loose and could be easily exfoliated. The internal rust layer with compact structure was closely connected with the reinforced bar matrix. The rust powder was grinded separately in grinding bowls until it passed through 330 mesh sieves. As shown in Figure 3, the test process involved first placing the proper amount of rust powder (about 2 g) on the supporting slide, smoothing the surface, and then placing the sample into the X-ray powder diffractometer (XRD-6000, Shimadzu Company, Kyoto, Japan). After XRD analysis, the test results were output from the computer and analyzed by software MDI Jade 6.5

#### 2.3.2. SEM Analysis

**Figure 4:**
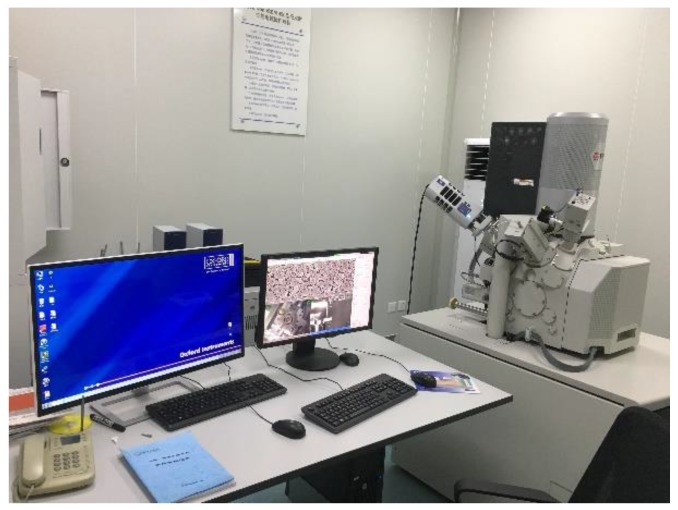
Nova NanoSEM450 scanning electron microscope.

After the specimens were broken, the concrete was hammered into soybean-sized particles. Then, 1-2 pieces of cement paste flake particles were selected on the surface of steel bars with corrosion products on the outside of steel bars. The specimens were packed in sealed bags under vacuum to prevent the corrosion products from continuing to oxidize in contact with air. The prepared samples were analyzed by Nova NanoSEM450 field emission scanning electron microscope (Waltham, MA, USA) (Figure 4). Corrosion products were observed three times at the same location. The magnification was 2000, 5000 and 10,000 times, respectively.

## 3. Results and Discussion

### 3.1. Product Analysis of Steel Rust Layer

**Figure 5:**
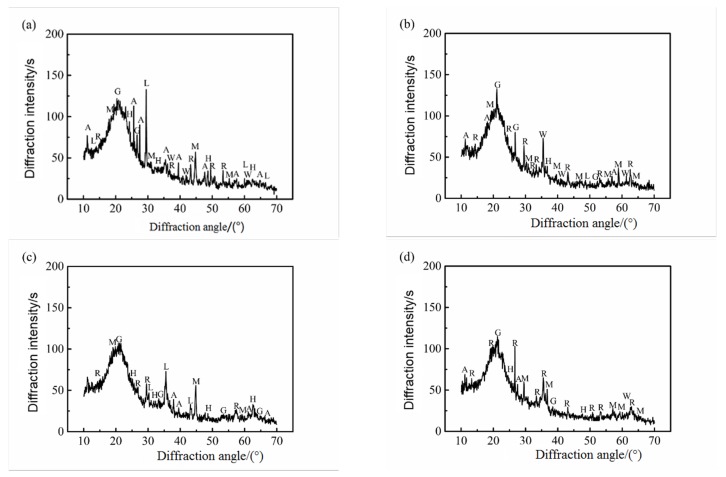
XRD patterns of rust sample of steel bar in seawater sea-sand concrete; (**a**) Inner rust layer of A1, (**b**) Outer rust layer of A1, (**c**) Inner rust layer of A2, (**d**) Outer rust layer of A2.

**Figure 6:**
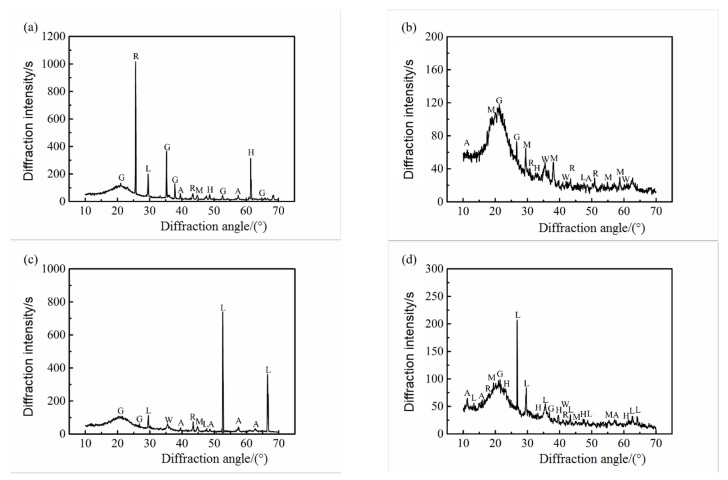
XRD patterns of rust sample of steel bar in ordinary concrete; (**a**) Inner rust layer of B1, (**b**) Outer rust layer of B1, (**c**) Inner rust layer of B2, (**d**) Outer rust layer of B2.

X-ray diffraction spectra of powder samples of the inner rust layer and the outer rust layer of carbon steel rust products in the two concretes are shown in Figure 5 and Figure 6. It can be seen from the two graphs that the corrosion products of carbon steel were a mixture of wustite (FeO), aka-ganeite (β–FeOOH), lepidocrocite (γ–FeOOH), goethite (α–FeOOH), magnetite (${\mathrm{Fe}}_{3}O_{4}$), maghemite (γ–${\mathrm{Fe}}_{2}O_{3}$), and hematite (α–${\mathrm{Fe}}_{2}O_{3}$). Among them, the diffraction peaks of maghemite (γ–${\mathrm{Fe}}_{2}O_{3}$), hematite (α–${\mathrm{Fe}}_{2}O_{3}$), lepidocrocite (γ–FeOOH), and goethite (α–FeOOH) were higher, indicating that the content of these rust products was higher, or their crystallinity was higher. In addition, some impurities of cement hydration products such as orthoclase, calcium silicate hydrate, calcium aluminate hydrate, and iron were also present in the samples, possibly due to the mutual infiltration of rust products and cement paste.

**Figure 7:**
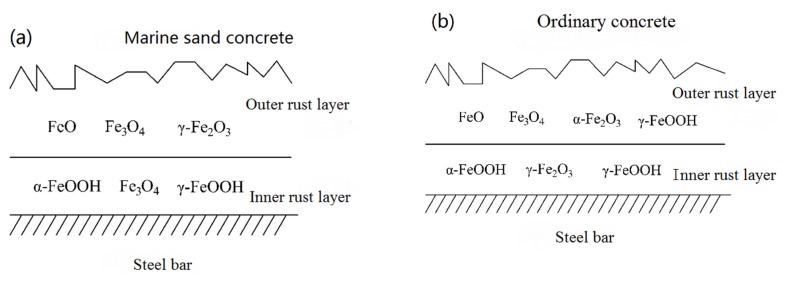
Schematic illustration of layered structure of rebar rust in different concrete; (**a**) Seawater and sea-sand concrete, (**b**) Ordinary concrete.

From Figure 5, it can be seen that the outer rust layer of steel bars in seawater and sea-sand concrete was mainly composed of wustite (FeO), magnetite (${\mathrm{Fe}}_{3}O_{4}$), and maghemite (γ–${\mathrm{Fe}}_{2}O_{3}$), while the inner rust layer was mainly composed of a mixture of goethite (α–FeOOH), magnetite (${\mathrm{Fe}}_{3}O_{4}$), and lepidocrocite (γ–FeOOH). Correspondingly, it can be seen from Figure 6 that under the condition of chloride corrosion, the outer rust layer of steel bar was mainly composed of magnetite (${\mathrm{Fe}}_{3}O_{4}$), hematite (α–${\mathrm{Fe}}_{2}O_{3}$), wustite (FeO) and lepidocrocite (γ–FeOOH), while the inner rust layer was mainly composed of a mixture of lepidocrocite (γ–FeOOH), goethite (α–FeOOH) and maghemite (γ–${\mathrm{Fe}}_{2}O_{3}$). At the same time, under the same sample treatment, sample quality, and sample conditions, the diffraction peak intensity of the outer rust layer was obviously lower than that of the inner rust layer, which indicates that the crystallinity of the corrosion products of the inner rust layer was better and the protection of the reinforced bar was stronger. On the other hand, the reason the corrosion rate of the steel bar decreases gradually in the later period was explained. The structural model of steel bar corrosion caused by sea water in seawater and sea-sand concrete is shown in Figure 7a and that of steel bar corrosion caused by sea water in ordinary concrete is shown in Figure 7b.

Some scholars believe that green rust hydrochloride ${\mathrm{GRCl}}^{-}({\left[{\mathrm{Fe}}_{3}^{2+}{\mathrm{Fe}}^{3+}{\left({\mathrm{OH}}^{-}\right)}_{8}\right]}^{+}\cdot {\left[{\mathrm{Cl}}^{-}\cdot {\mathrm{nH}}_{2}O\right]}^{-}$*)* is very unstable at the interface between concrete and steel matrix, and easily oxidizes into new corrosion products aka-ganeite (β–FeOOH). Misawa and others [24] reported that lepidocrocite (γ–FeOOH) and goethite (α–FeOOH) are denser than aka-ganeite (β–FeOOH) with monoclinic cell structure. Moreover, because ${\mathrm{Cl}}^{-}$ and ${\mathrm{OH}}^{-}$ can stablize the pipeline substructure, water can be retained in the gap between aka-ganeite (β–FeOOH) acicular crystals, providing favorable conditions for carbon steel to aggravate pitting corrosion. At the same time, ${\mathrm{Cl}}^{-}$ can replace the ${\mathrm{OH}}^{-}$ radical of (β–FeOOH) and form a new corrosion product phase β–FeO(OH,Cl).

Previous studies [25,26] have found that some components of corrosion products of steel bars can act as new depolarizers in cathodic reduction reaction without oxygen. This explains the continuous corrosion of steel bars in long-term immersed specimens. Samples A1 and B1 were immersed in seawater for a long time. Although oxygen supply was limited, the substitution in the rust layer of carbon steel became a new depolarizer. Thus, cathodic reduction reaction occurred at the interface where the resulting corrosion could be described by the following equation [27]:

(1) $$6\mathrm{FeOOH}+2e^{-}\to 2{\mathrm{Fe}}_{3}O_{4}+2H_{2}O+2{\mathrm{OH}}^{-}$$

The above chemical reaction not only solves the stagnation of cathodic reduction reaction caused by lack of oxygen, but also reduces the resistance of hydrated ${\mathrm{Fe}}^{2+}$ in the transmission process with the dissolution of FeOOH attached to the steel matrix. It can be concluded that the solution layer near the surface of steel bar contained high concentration of ${\mathrm{Fe}}^{2+}$. Without the barrier effect of FeOOH, the anodic iron dissolved rapidly, and the anodic polarization of carbon steel decreased rapidly. Consequently, the potential difference decreased rapidly, and the corrosion process of steel bar continued. Moreover, the saturation rate of specimens is inversely proportional to the resistivity of concrete. The corrosion current of steel bar under immersion condition was larger than that under dry-wet cycling condition. With the progress of corrosion process, FeOOH, as a cathode depolarizer under anoxic conditions, was gradually depleted. At this time, a large number of ${\mathrm{Fe}}_{2}O_{3}$ and ${\mathrm{Fe}}_{3}O_{4}$ corrosion products were accumulated on the surface of steel bars, and the corrosion current decreased gradually until the corrosion process stopped.

### 3.2. Microstructure Analysis of Steel Rust Layer

**Figure 8:**
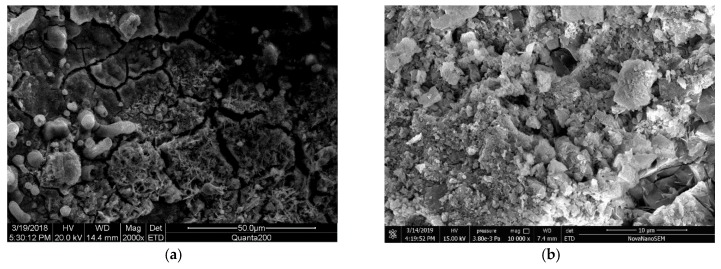
SEM images of rust layer in seawater and sea-sand concrete under continuous immersion; (**a**) Initial rust layer morphology of A1, (**b**) Later rust layer morphology of A1.

**Figure 9:**
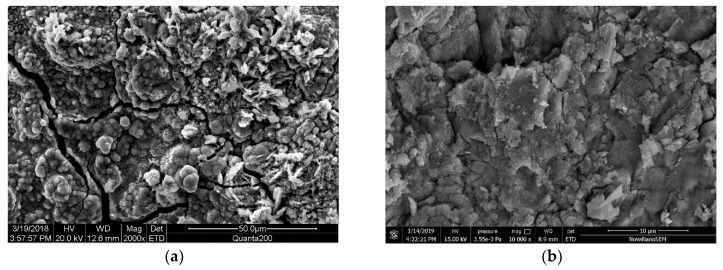
SEM images of rust layer in seawater and sea-sand concrete under dry-wet cycle; (**a**) Initial rust layer morphology of A2, (**b**) Later rust layer morphology of A2.

**Figure 10:**
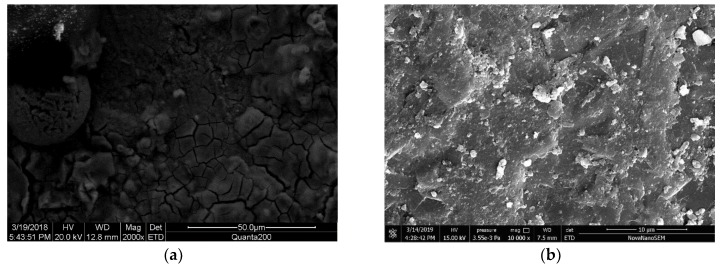
SEM images of rust layer in ordinary concrete under continuous immersion; (**a**) Initial rust layer morphology of B1, (**b**) Later rust layer morphology of B1.

**Figure 11:**
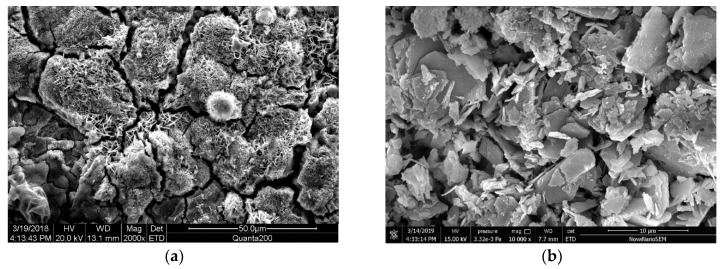
SEM images of rust layer in ordinary concrete under dry-wet cycle; (**a**) Initial rust layer morphology of B2, (**b**) Later rust layer morphology of B2.

To understand the morphological characteristics of the corrosion layer of steel bars, samples A1, B1, A2 and B2 were broken and sampled, and the morphology of the corroded layer was observed by SEM. Figure 8, Figure 9, Figure 10 and Figure 11 show the SEM images of the morphology of corrosion products of carbon steel in various types of concrete in different periods. It can be seen that the corrosion products exhibited different morphologies, such as rod, needle, scale, granular, and block. The corrosion products were integrated with the cross-network corrosion products attached to the surface of the steel bar. Based on the XRD results, it can be inferred that the large-sized products were magnetite (${\mathrm{Fe}}_{3}O_{4}$), needle-like or rod-like products were goethite (α–FeOOH), the cross-network corrosion products were aggregates of lepidocrocite (γ–FeOOH) and goethite (α–FeOOH), while the spherical, scaly and granular products were mixtures of hematite (α–${\mathrm{Fe}}_{2}O_{3}$) and maghemite (γ–${\mathrm{Fe}}_{2}O_{3}$). α–FeOOH can adhere to the surface of steel bar, so the dissolution rate of anodic iron decreased. This is the main reason the corrosion rate of steel bar decreased gradually with the progress of corrosion. According to the elemental composition determined by EDS (Table 3), the corrosion products included not only iron oxides, but also cement hydration products such as orthoclase, calcium silicate hydrate, and calcium aluminate hydrate. This was likely due to the infiltration of corrosion products into cement paste.

From Figure 8, it can be seen that the corrosion products of steel bars in seawater sand concrete specimens exhibited massive and granular morphology at the initial and later stages, but the structure was relatively loose. Moreover, the corrosion products formed in seawater sand concrete specimens under immersion conditions were mostly black and some were brown–red in color. Combined with the XRD results, it can be inferred that the corrosion product was mainly composed of wustite FeO, magnetite (${\mathrm{Fe}}_{3}O_{4}$) and lepidocrocite (γ–FeOOH), where wustite FeO was the major component and was formed by the reaction of rust intermediates. The related reaction is as follows:

(2) $$\mathrm{Fe}{\left(\mathrm{OH}\right)}_{2}\to \mathrm{FeO}+H_{2}O$$

Due to the long-term immersion of the specimens, it was difficult for oxygen to reach the surface of steel bars during the corrosion process. Thus, the other part of the specimens was oxidized incompletely and became black magnetite (${\mathrm{Fe}}_{3}O_{4}$). The reaction formula is as follows:

(3) $$6\mathrm{Fe}{\left(\mathrm{OH}\right)}_{2}+O_{2}\to 2{\mathrm{Fe}}_{3}O_{4}+6H_{2}O$$

Figure 9 shows the SEM morphology of the early and later corrosion products of carbon steel in seawater sand concrete specimens under dry-wet cycling. It can be seen from the images that the initial corrosion products were mostly granular aggregates with compact texture, including some acicular and flaky products, which are typical maghemite (γ–${\mathrm{Fe}}_{2}O_{3}$). Later, the corrosion products of steel bars were similar to those of Figure 8a, both massive and granular, but the structure of corrosion products in this image was more compact. Moreover, the corrosion products of seawater sand concrete specimens under dry-wet cycling conditions were mainly ferrous black and brown in color. Combined with the XRD results, it was inferred that the content of magnetite (${\mathrm{Fe}}_{3}O_{4}$) and maghemite (γ–${\mathrm{Fe}}_{2}O_{3}$) was higher in the corrosion product. According to Legrand’s theory, because seawater contains a large amount of sulfate, it reacts with $\mathrm{Fe}{\left(\mathrm{OH}\right)}_{2}$ to produce green rust hydrochloride ${\mathrm{GRCl}}^{-}\left({\left[{\mathrm{Fe}}_{3}^{2+}{\mathrm{Fe}}^{3+}{\left({\mathrm{OH}}^{-}\right)}_{8}\right]}^{+}\cdot {\left[{\mathrm{Cl}}^{-}\cdot {\mathrm{nH}}_{2}O\right]}^{-}\right)$ first, and green rust hydrochloride ${\mathrm{GRCl}}^{-}\left({\left[{\mathrm{Fe}}_{3}^{2+}{\mathrm{Fe}}^{3+}{\left({\mathrm{OH}}^{-}\right)}_{8}\right]}^{+}\cdot {\left[{\mathrm{Cl}}^{-}\cdot {\mathrm{nH}}_{2}O\right]}^{-}\right)$ continues to oxidize to produce orange-yellow lepidocrocite (γ–FeOOH). Due to the frequent dry-wet cycles in this experiment, the environment of the sample was wet. Thus, lepidocrocite (γ–FeOOH) was transformed into magnetite (${\mathrm{Fe}}_{3}O_{4}$), and magnetite (${\mathrm{Fe}}_{3}O_{4}$) finally formed maghemite (γ–${\mathrm{Fe}}_{2}O_{3}$) through secondary changes under sufficient oxygen conditions. The related reaction equations are as follows:

(4) $${\mathrm{Fe}}^{2+}+8\gamma –\mathrm{FeOOH}+2e^{-}\to 3{\mathrm{Fe}}_{3}O_{4}+4H_{2}O$$

(5) $$2{\mathrm{Fe}}_{3}O_{4}+5O_{2}\to 3\gamma –{\mathrm{Fe}}_{2}O_{3}$$

Figure 10 presents the SEM images of the corrosion products of carbon steel in ordinary concrete specimens under immersion conditions. It can be seen from the images that the corrosion products of steel bar in the early stage were block magnetite (${\mathrm{Fe}}_{3}O_{4}$) and spherical hematite (α–${\mathrm{Fe}}_{2}O_{3}$). Goethite (α–FeOOH) was strongly adhered to the surface of steel bar, which reduced the dissolution rate of anodic iron. This is also the reason the corrosion rate of steel bar decreased gradually with the development of corrosion. In the later stage, the morphology of corrosion products was similar to that in Figure 10a, but the texture was more compact, and the granular material was less. Moreover, the corrosion products of ordinary concrete specimens under immersion conditions were mostly black in color. Combined with the XRD results, it was inferred that magnetite (${\mathrm{Fe}}_{3}O_{4}$) was the main product, which contained some partially powdered wustite (FeO) from incomplete oxidation.

Figure 11 presents the SEM morphology of the corrosion products of carbon steel in common concrete specimens under dry-wet cycling conditions. It can be seen that in the early stage, the corrosion products contained many needle–spherical aggregates, which were adhered to the surface of lepidocrocite (γ–FeOOH) and goethite (α–FeOOH). In the later stage, the corrosion products of reinforcing bars were mostly scaly, fibrous and granular, and the structure of the products was loose. Comparing the color of the corrosion products, the corrosion products of ordinary concrete specimens under wet/dry cycling conditions were mainly brown–red and some of them were orange-yellow, indicating typical hematite (α–${\mathrm{Fe}}_{2}O_{3}$) and lepidocrocite (γ–FeOOH) mixed products. In the wet-dry cycle, the oxygen supply was enough, so the Fe element was fully oxidized into trivalent iron ions. Lepidocrocite (γ–FeOOH) was converted to maghemite (γ–${\mathrm{Fe}}_{2}O_{3}$) under dehydration condition, and maghemite (γ–${\mathrm{Fe}}_{2}O_{3}$) was converted to hematite (α–${\mathrm{Fe}}_{2}O_{3}$). At the same time, some lepidocrocite (γ–FeOOH) was converted to goethite (α–FeOOH) under certain conditions, and goethite (α–FeOOH) was converted to final product hematite (α–${\mathrm{Fe}}_{2}O_{3}$) under dehydration condition. The related reactions are as follows:

(6) $$2\gamma –\mathrm{FeOOH}\to \gamma –{\mathrm{Fe}}_{2}O_{3}+H_{2}O$$

(7) $$2\alpha –\mathrm{FeOOH}\to \alpha –{\mathrm{Fe}}_{2}O_{3}+H_{2}O$$

According to the results of XRD and SEM analyses, combined with the oxidation mode of concrete specimens, it can be speculated that goethite (α–FeOOH) and lepidocrocite (γ–FeOOH) dehydrate and transform into hematite (α–${\mathrm{Fe}}_{2}O_{3}$) after dehydration. Once the environment becomes damp, hematite (α–${\mathrm{Fe}}_{2}O_{3}$) can also absorb water and convert into goethite (α–FeOOH). γ–FeOOH can easily convert into more stable magnetite (${\mathrm{Fe}}_{3}O_{4}$). Magnetite (${\mathrm{Fe}}_{3}O_{4}$) can be reconverted to maghemite (γ–${\mathrm{Fe}}_{2}O_{3}$) and further oxidized to more stable goethite (α–FeOOH) by secondary changes under oxidation conditions. With the diffusion of oxygen and water vapor, new lepidocrocite (γ–FeOOH) is constantly generated. Thus, the corrosion reaction constantly progresses, and the thickness of the rust layer increases continuously.

## 4. Conclusions

In this paper, the material composition, the intrinsic morphology and the secondary electronic signal imaging of steel corrosion products in seawater and sea-sand concrete and ordinary concrete under different curing methods were analyzed by XRD and SEM, and the corrosion products of steel in two kinds of concrete in different periods were determined. Based on the test results of the specimens, the formation and development process of corrosion products of steel bars under different corrosion modes and the development mechanism were analyzed. The main conclusions of this work are as follows:

(1) The passive film of reinforcing bar in concrete was destroyed mainly by electrochemical corrosion reaction. Under the action of oxygen and water, iron lost electrons and metal anodic dissolution occurred. The electrons obtained from oxygen and water underwent cathodic reduction reaction of depolarizer, resulting in the intermediate product $\mathrm{Fe}{\left(\mathrm{OH}\right)}_{2}$ of reinforcing bar corrosion.

(2) Due to the large amount of chloride ions present in the aggregate and mixing water of seawater sand concrete, the internal rust layer contained many transition products, including lepidocrocite (γ–FeOOH) and aka-ganeite (β–FeOOH).

(3) Under wet/dry cycling conditions, steel corrosion products tended to form maghemite (γ–${\mathrm{Fe}}_{2}O_{3}$) and hematite (α–${\mathrm{Fe}}_{2}O_{3}$) due to dehydration, while hematite (α–${\mathrm{Fe}}_{2}O_{3}$) absorbed water and converted to goethite (α–FeOOH) under specific conditions. That is to say, with the supplement and loss of water in concrete, various corrosion products were dynamically transformed into each other.

(4) The content of lepidocrocite (γ–FeOOH) and aka-ganeite (β–FeOOH) observed in seawater samples for a long time was relatively small. The reason is that they can replace $O_{2}$ and $H_{2}O$ as depolarizers of cathodic reduction reaction without oxygen and consume themselves to ensure smooth corrosion. 

## Figures and Tables

**Table 1:** Composition of steel (%).

Chemical Composition	C	Si	Mn	P	S
η (%)	0.16	0.24	0.46	0.032	0.029

**Table 2:** Test specimen grouping table.

Specimen Name	Types of Concrete	Curing Methods
A1	Seawater and sea-sand concrete	immersion
A2	Seawater and sea-sand concrete	wet/dry cycles
B1	Ordinary concrete	immersion
B2	Ordinary concrete	wet/dry cycles

**Table 3:** Elemental composition of specimens measured by EDS (%).

Specimen Name	O	Si	Ca	Fe
A1	51.16	2.42	3.75	42.66
A2	35.12	1.32	4.82	58.74
B1	39.88	1.01	8.22	50.88
B2	38.51	3.46	8.90	49.13
